# USE OF TECHNOLOGY TO DELIVER AND MONITOR NONPHARMACOLOGICAL INTERVENTION FOR ADRD WITH BEHAVIORAL SYMPTOMS

**DOI:** 10.1093/geroni/igac059.1586

**Published:** 2022-12-20

**Authors:** Elizabeth Rhodus, Rosmy George, Richard Kryscio, Justin Barber, Allison Gibson, Donna Wilcock, Gregory Jicha

**Affiliations:** University of Kentucky, Lexington, Kentucky, United States; University of Kentucky, Lexington, Kentucky, United States; University of Kentucky, Lexington, Kentucky, United States; University of Kentucky, Lexington, Kentucky, United States; University of Kentucky, Lexington, Kentucky, United States; University of Kentucky, Lexington, Kentucky, United States; University of Kentucky, Lexington, Kentucky, United States

## Abstract

Onset of the COVID-19 pandemic created numerous barriers to providing care supports and clinical research for persons living with Alzheimer’s disease and related dementias (ADRD). Technology offered one avenue for continued care support and clinical data collection. This study reports on the use of technology to deliver a 6-week, non-pharmacological care intervention directed toward caregivers of persons with ADRD and remote data collection including cognitive assessment, biometric data, and survey data for community-residing persons with ADRD and behavioral symptoms (N=28). Benefits and challenges of such technology use for intervention delivery and data collection will be discussed. Benefits include increased geographical outreach, no travel time, and greater scheduling flexibility. Challenges include access to technology (equipment and/or internet), internet connection quality, ease of use, and equipment return at study completion. These findings offer specific aspects to consider while designing and implementing remote care programming and clinical research for community-residing persons with ADRD.

